# Origins and characteristics of dissolved organic matter fueling harmful dinoflagellate blooms revealed by δ^13^C and d/l-Amino acid compositions

**DOI:** 10.1038/s41598-022-19168-7

**Published:** 2022-09-05

**Authors:** Jihyun Park, Guebuem Kim, Hyeong Kyu Kwon, Heejun Han, Tae Gyu Park, Moonho Son

**Affiliations:** 1 School of Earth and Environmental Sciences/Research Institute of Oceanography, Seoul National University, Seoul, 08826 Republic of Korea; 2 Southeast Sea Fisheries Research Institute, National Institute of Fisheries Science, Tongyeong, 53085 Republic of Korea; 3 Ocean Climate and Ecology Research Division, National Institute of Fisheries Science, Busan, 46083 Republic of Korea

**Keywords:** Biogeochemistry, Marine chemistry

## Abstract

We measured the concentrations of dissolved inorganic and organic nutrients, dissolved organic carbon (DOC), total hydrolyzable amino acids (THAA), fluorescent dissolved organic matter (FDOM), phytoplankton pigments, and δ^13^C-DOC during the summer of 2019 in the harmful dinoflagellate bloom regions of the southern coast of Korea. In the harmful dinoflagellate bloom region, the concentrations of inorganic nitrogen were depleted, inhibiting the growth of diatoms, while the concentrations of dissolved organic components (nutrients, DOC, FDOM, and amino acids) which fuel dinoflagellates were unusually high. Thus, we attempted to investigate the origins and characteristics of DOM which fuels the harmful dinoflagellate blooms. The δ^13^C-DOC values (− 22.2‰ to − 18.2‰) indicate that the elevated DOC concentrations result from in-situ biological production rather than terrestrial inputs. The enantiomeric (D/L) ratios of THAA indicate that dissolved organic nitrogen was more labile in the early stage of harmful dinoflagellate bloom and became more refractory in the final stage. Our results suggest that the marine production of bioavailable DOM plays an important role in initiating and sustaining harmful dinoflagellate blooms.

## Introduction

The outbreak of harmful algal blooms in the coastal ocean associated with increased inputs of nutrients has been reported worldwide^1,2^. In particular, dinoflagellate blooms often have serious effects on aquaculture and wild organisms as they cause massive kills of fish and other invertebrate by producing toxins or mucus substances^3,4^. The environmental conditions in harmful dinoflagellate bloom (HDB) regions have been characterized by low concentrations of dissolved inorganic nutrients and high concentrations of dissolved organic nutrients^5–8^. Many culture experiments and field observations showed that this condition is favorable for the growth of dinoflagellates in competition with diatoms since HDB forming species (e.g., *Margalefidinium*, *Alexandrium*) are capable of converting organic nitrogen compounds to inorganic nutrients, while the growth of diatoms is limited under low inorganic nutrient concentrations^8–13^.

Many studies were conducted to determine the characteristics of dissolved organic matter (DOM) in HDB regions. The concentrations of dissolved organic carbon (DOC) in the bloom regions were significantly higher than those of non-bloom regions in Yeosu and Tongyeong of Korea^14,15^. The concentrations of fluorescent dissolved organic matter (FDOM) which is commonly composed of marine humic-like (FDOM_M_), terrestrial humic-like (FDOM_C_), and protein-like (FDOM_T_) components also increased in HDB areas^14,16,17^. FDOM, which contributes about 20–70% of the DOC in coastal waters^18^, is known to play an important role in the outbreak of HDBs as it protects organisms from UV radiation and becomes an energy source for the growth of dinoflagellates^18–20^.

In HDB regions, DOM originates from various sources including rivers and groundwater as well as in-situ production^15,21^. In general, humic-like FDOM originate mainly from terrestrial sources^18,22^, although Kwon et al.^14^ showed in-situ production of FDOM_C_ in HDB regions. FDOM_T_, which is mostly labile, is produced freshly by biological activities in HDB regions^16,23^. Although DOM is known to fuel dinoflagellates in HDB regions^7,11^, the chemical compositions and characteristics of DOM have been still poorly understood.

Thus, we attempted to determine the sources and characteristics of DOC and dissolved organic nitrogen (DON) in the HDB region of Korea. We measured various biogeochemical parameters, together with carbon stable isotope ratios of dissolved organic carbon (δ^13^C-DOC) and l- and d-amino acids, in the southern sea of Korea, during the 2019 summer when a HDB occurred. We hypothesized that δ^13^C-DOC could discriminate DOM sources between marine origins (− 18‰ to − 22‰) and terrestrial origins (− 23‰ to − 34‰ from C_3_ plants and − 9‰ to − 17‰ from C_4_ plants)^24,25^ and that d- and l-amino acid compositions can determine the bioavailability of DON in the HDB region^26,27^. Based on the molar composition or relative abundance of amino acids (e.g., enantiomeric ratio of amino acids, nitrogen-normalized yield of amino acids), several diagenetic indicators have been utilized to determine the biodegradation state of organic matter^28–30^.

## Materials and methods

### Study area

**Figure 1:**
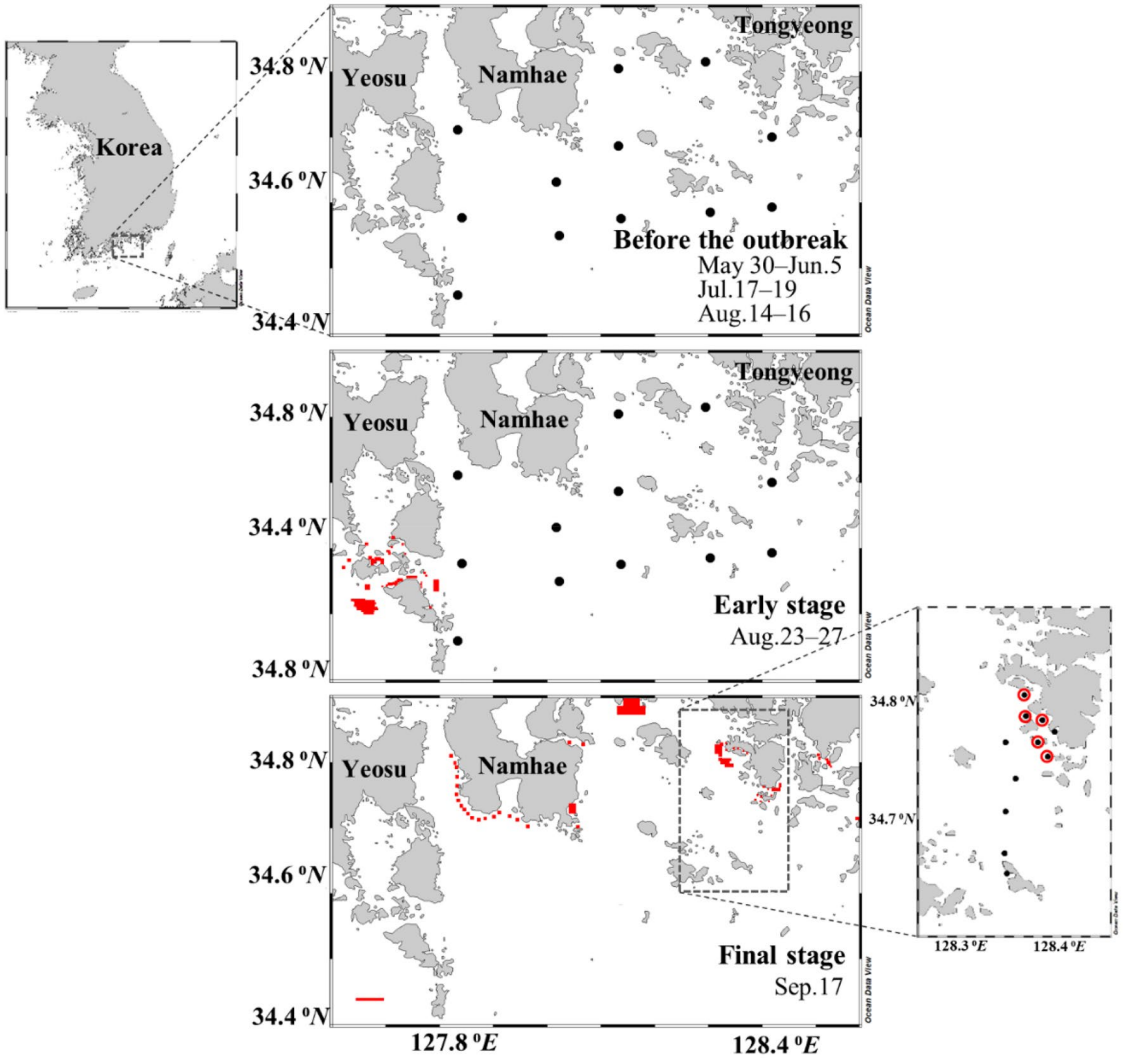
Map of the sampling stations (black circles) during May 30–June 5, July 17–19, and August 14–16 (before the outbreak of red tide), August 23–27 (the early stage of red tide), and September 17 (the final stage of red tide), 2019, in the southern coast of Korea. The red dots and the red circles represent the red tide patches (http://www.nifs.go.kr/)^40^.

The study area is located in a coastal region off the southern coast of Korea (34° 39′ 12.65″ N–34° 48′ 25.16″ N, 128° 20′ 23.99″ E–128° 23′ 32.31″ E) (Fig. 1). The water depth of this region is shallower than 10 m in nearshore areas, and reaches up to 50 m in offshore areas. The seawater of this region is highly affected by the oligotrophic Tsushima Current, which is a branch of the Kuroshio Current^31–33^. The Changjiang Diluted Water (CDW), which is carried by the Tsushima Current, often influences this region, particularly during summer and fall^34^. However, the nutrients in the CDW-influenced waters can be completely consumed before entering the study region^33^, and most of the dissolved total nitrogen (DTN) in the study region is known to originate from the local sources^5,8^. In addition, the Seomjin River (drainage area: 4896 km^2^) influences the salinity and the nutrient concentrations in this region, mainly during the summer monsoon season.

In the southern coast of Korea, *Margalefidinium polykrikoides* (formerly *Cochlodinium polykrikoides*) is the dominant species causing HDBs (hereafter red tides)^35^. In this region, red tides have occurred repeatedly since 1982. In the southern sea of Korea, the inputs of dissolved inorganic nitrogen (DIN) from heavy rainfall events^36^ and the Yangtze River diluted water^37^ had been attributed to the main sources of nutrients. However, Lee and Kim^5^ showed that local sources, such as submarine groundwater discharge, were the main source of nutrients fueling red tides. The rapid growth of *M. polykrikoides* in competition with diatoms happened under the condition of depleted DIN or dissolved inorganic phosphorus (DIP) and enriched organic nutrients, when the supply of inorganic nutrients was halted^5,6,8,38,39^. In general, *M. polykrikoides* patches formed in offshore waters are transported to nearshore waters and gradually accumulated as the abundance of competing phytoplankton species (i.e., fast-growing diatoms) is low^13^.

In the summer of 2019, *M. polykrikoides* red tides were first observed in the study region on August 23 and then spread to the wide area of the study region (http://www.nifs.go.kr/)^40^. The red tide disappeared around September 24. The seawater temperatures of the study area ranged from 23 to 26 °C during the red tide outbreak periods, which were within the optimum temperature range (21–26 °C) for the growth of *M. polykrikoides*^41^. The salinity ranged from 30 to 34 psu throughout the study period. The patches of red tide were mostly located in proximity to the shore, perhaps associated with passive accumulation by geographical and tidal features in this region (Fig. 1).

### Sampling

Seawater sampling was conducted in 2017, 2018, and 2019. Red tide outbreak was observed in 2018 and 2019. In 2017, sampling was performed from July 31 to August 1. In 2018, seawater samples were collected from July 30 to August 3 during the red tide period. In 2019, sampling was conducted before the outbreak (May 30–June 5, July 17–19, and August 14–16, 2019) and during the early stage of red tide occurrence (August 23–27, 2019) at 12 sites (Fig. 1). Additional field observations were conducted in a small region of Tongyeong (11 sites) when the red tide had declined (September 17, 2019).

Seawater samples were collected in the surface layer (~ 0.5 m depth) using a submersible pump on shipboard from 2017 to 2019. Samples for DOC and FDOM were collected for all the sampling periods. Additionally, in 2019, samples for dissolved inorganic/organic nutrients were collected from May to September. Samples for δ^13^C-DOC and amino acids were collected over three periods in 2019 (before the outbreak: August 14–16, the early stage: August 23–27, and the final stage: September 17). Samples for the analysis of phytoplankton pigments were collected during the final stage of red tide (September 17, 2019). Salinity and temperature were measured in-situ using a portable multimeter (Orion Star A329, Thermo Scientific, USA). All seawater samples for the dissolved form were filtered through pre-combusted (450 °C, 5 h) glass-fiber filters (Whatman GF/F, pore size: 0.7 µm). The filter samples were used to measure the concentrations of phytoplankton pigments. Sample analyses were completed mostly within a week from the sample collection.

### Analyses

#### Phytoplankton pigment analyses

The filter samples for pigment analysis were stored at − 80 °C. The filter samples were extracted in 95% methanol, with an internal standard (canthaxanthin), at 4 °C for 24 h in the dark. Extracts were sonicated (5 min) and centrifuged (3500 rpm, 10 min) and then filtered through PTEE membrane filters (pore size: 0.2 µm) to remove residual particles. The extracts were analysed for pigments by using high-performance liquid chromatography (HPLC) (Waters 2695) with a Waters Symmetry C8 column (4.6 × 150 mm, particle size: 3.5 μm, pore size: 100 Å) according to the method described by Zapata et al.^42^. Identification and quantification of chlorophyll *a* (chl. *a*), fucoxanthin (marker pigment for diatom), and peridinin (marker pigment for dinoflagellate) were based on their retention times with authentic standards (DHI Inc., Denmark).

#### Nutrient analyses

The seawater samples for nutrient analysis were stored at − 20 °C. The concentrations of DIP (PO_4_^3−^) and DIN (the sum of NH_4_^+^, NO_3_^−^, and NO_2_^−^) were measured by an auto nutrient analyzer (New QuAAtro39, Seal Analytical, Germany). The analytical accuracy was verified with reference seawater materials (KANSO Technos, Japan). Dissolved total phosphorus (DTP) and DTN were analysed using the same instrument after chemical oxidation by potassium persulfate at 120 °C for 30 min^43^. The concentrations of dissolved organic phosphorus (DOP) and DON were calculated by subtracting the measured DIP and DIN from the measured DTP and DTN concentrations, respectively.

#### FDOM analyses

The seawater samples for FDOM were stored at 4 °C in pre-combusted (450 °C, 5 h) amber vials. The FDOM was measured by a spectrofluorometer (Aqualog, Horiba, USA). The scanning wavelength for fluorescence excitation-emission matrices (EEMs) was 252–600 nm in 2 nm increments for excitation (Ex) and 211–616 nm in 3 nm increments for emission (Em) with an integration time of 3 s. Raman and Rayleigh scattering signals, the inner-filter effect, and blank subtraction were corrected using the SOLO software (Eigenvector Research Inc., WA, USA). The fluorescence intensity was normalized by the Raman peak area of ultrapure water and referred to as Raman units (R.U.)^44^. The FDOM analyses with the parallel factor analysis (PARAFAC) model identified one protein-like fluorescence peak and two humic-like fluorescence peaks, as described by Coble^18^. Component 1 (Ex/Em = 278/340 nm), component 2 (Ex/Em = 362/466 nm), and component 3 (Ex/Em = 338/400 nm) were found to be FDOM_T_ (tryptophan-like), FDOM_C_, and FDOM_M_, respectively. The PARAFAC model results were validated by random initialization and split-half analysis^45^.

#### *DOC and δ*^*13*^*C-DOC analyses*

The seawater samples for DOC and δ^13^C-DOC were stored at room temperature in pre-combusted glass ampoules (450 °C, 5 h) after being acidified with 6 M HCl. The DOC concentration was determined by a high-temperature catalytic oxidation (HTCO) method using a total organic carbon (TOC) analyzer (TOC-L, Shimadzu, Japan)^46,47^. Verification of analytical accuracy was performed using a reference material (~ 43 μM; University of Miami, USA). The δ^13^C-DOC values were measured by an isotope ratio mass spectrometer (IRMS; Isoprime, Elementar, Germany) connected to a TOC analyzer (Vario TOC cube, Elementar, Germany) via an interface system (isoTOC interface, Elementar, UK)^48^. The δ^13^C-DOC values were verified with the reported values of the reference materials: IAEA-CH6 sucrose (δ^13^C = − 10.45 ± 0.03‰), Suwannee River fulvic acid (δ^13^C = − 27.6 ± 0.12‰; International Humic Substances Society), and deep-seawater reference (University of Miami, USA) as previously stated in Lang et al.^49^ (δ^13^C = − 21.7 ± 0.3‰) and Panetta et al.^50^ (δ^13^C = − 21.4 ± 0.3‰).

#### Amino acid analyses

The seawater samples for amino acids were stored at − 20 °C. The d- and l-enantiomers of amino acids were analysed as described by Dittmar et al.^51^. The seawater samples were hydrolyzed with 12 M HCl and 11 mM ascorbic acid at 110 °C for 24 h after flushing with ultra-pure nitrogen. After hydrolysis, amino-acid enantiomers were derivatized with *o*-phthaldialdehyde and *N*-isobutyryl-l-cysteine. The derivatized samples were measured by a HPLC system equipped with an Alltima HP C18 column (particle size: 5 µm, 4.6 × 150 mm) and a Waters 2475 fluorescence detector (Ex/Em: 330/445 nm). A total of 13 individual amino acids were included in the analysis: serine (Ser), glutamic acid (Glu), aspartic acid (Asp), alanine (Ala), threonine, glycine, arginine, tyrosine, valine, phenylalanine, leucine, isoleucine, and γ-amino butyric acid (GABA). During the hydrolysis, glutamine and asparagine are converted to glutamic acid and aspartic acid, respectively. Therefore, Glu refers to the sum of glutamic acid and glutamine, and Asp refers to the sum of aspartic acid and asparagine. The concentrations of d-enantiomers and l- enantiomers of Ser, Glu, Asp, and Ala were averaged, respectively, for the d- and l-amino acid values in this study. The concentrations of total hydrolyzable amino acids (THAA) were taken as the sum of the 12 individual amino acids, except for the non-protein amino acid GABA. The nitrogen-normalized yields of THAA (THAA [%DON]) were calculated according to the method proposed by Davis and Benner^52^. A standard mixture of 13 individual amino acids was used for calibration and quantification (Sigma-Aldrich).

#### Statistical analyses

The one-way Analysis of Variance (ANOVA) with Scheffe’s test was conducted to evaluate the significance of the variations in nutrients and DOM for different sampling periods. Prior to the analysis, the normality and homogeneity of the variances were analysed using Shapiro–Wilk test and Levene’s test, respectively, to examine the validity of the ANOVA assumptions. When one of the assumptions was violated, Kruskal–Wallis test with Bonferroni test was conducted. To assess the difference in variations between the patch area and non-patch area during the red tide periods, *t*-test or Mann–Whitney *U* test (nonparametric) was conducted. All statistical analyses were conducted using SPSS 26 software for Windows (SPSS Inc., Chicago, IL). The significance level was set at *p* < 0.05 for all performed statistical tests.

## Results

**Figure 2:**
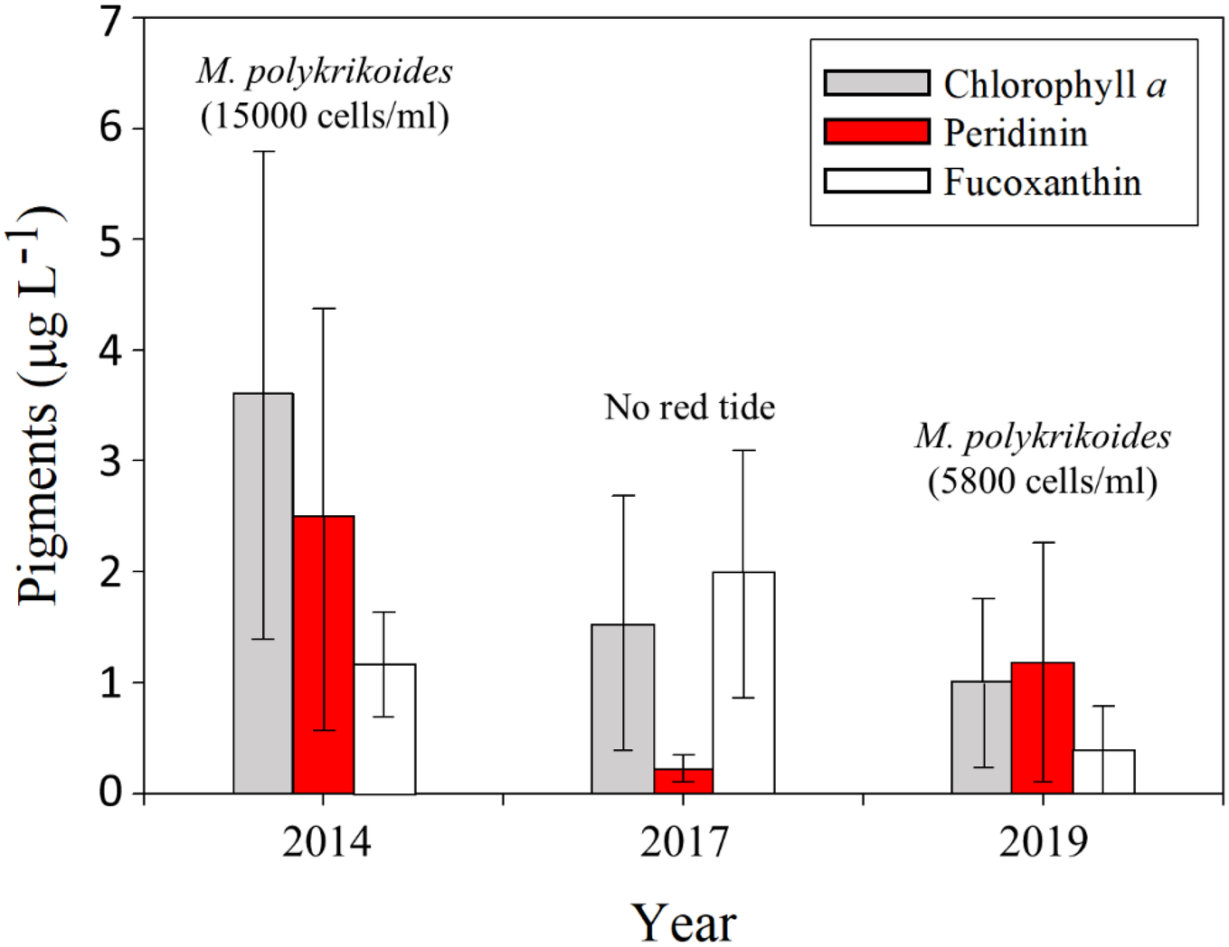
Average concentrations of chlorophyll *a*, peridinin, and fucoxanthin in coastal waters off Tongyeong, Korea, during the summers of 2014, 2017, and 2019. The maximum cell densities of *M. polykrikoides* were obtained from the National Institute of Fisheries Science (NIFS), Korea (http://www.nifs.go.kr/)^40^. The pigment concentrations in 2014 and 2017 in the red tide regions of Tongyeong were obtained from Kwon et al.^8^.

During the red tide period of September 17, 2019, the concentrations of chl. *a*, peridinin, and fucoxanthin were in the range of 0.1–2.3 µg L^−1^ (average: 1.0 ± 0.8 µg L^−1^), 0.03–3.3 µg L^−1^ (average: 1.2 ± 1.1 µg L^−1^), and 0.1–1.5 µg L^−1^ (average: 0.4 ± 0.4 µg L^−1^), respectively (Fig. 2). The average concentrations of chl. *a* and peridinin in the patch areas were 2.8- and 4.5-fold higher than those in the non-patch areas, respectively, while the concentrations of fucoxanthin in the patch areas were 0.7-fold lower than those in the non-patch areas (Supplementary Fig. S5).

**Figure 3:**
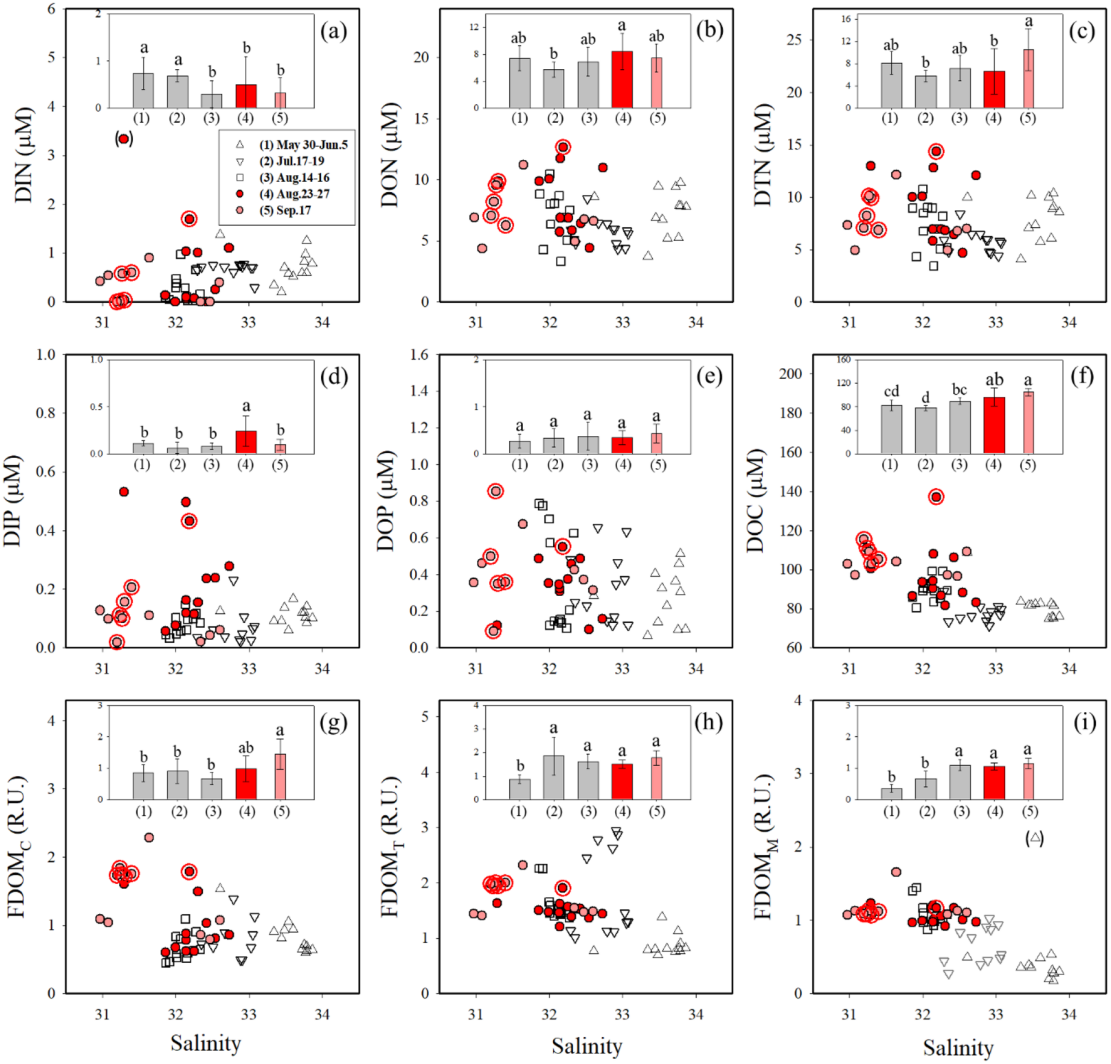
Scatter plots of salinities versus (**a**) DIN, (**b**) DON, (**c**) DTN, (**d**) DIP, (**e**) DOP, (**f**) DOC, (**g**) FDOM_C_, (**h**) FDOM_T_, and (**i**) FDOM_M_ in the Tongyeong coast during May 30–June 5, July 17–19, August 14–16, August 23–27, and September 17, 2019. The red circles represent the centers of the red tide patches. The vertical bars in the scatter plots show the average concentrations for each period. Significance (*p*) of the differences among the group (1, 2, 3, 4, and 5) in all figures was always < 0.05.

During all sampling periods in 2019 (before and after the red tide outbreak), the concentrations of DIN and DIP ranged from 0.0 to 3.3 µM and from 0.02 to 0.53 µM, respectively (Fig. 3). In general, the concentrations of DON (3.0–13.0 µM) and DOP (0.07–0.85 µM) were much higher than those of DIN and DIP for the same samples (Fig. 3). Overall, the concentrations of DIN during the red tide periods were significantly lower than those before the red‐tide outbreak periods (Kruskal–Wallis: *p* = 0.001) (Fig. 3). Although the average concentrations of DON in the patch areas (8.9 ± 2.3 µM) were slightly higher than those in the non-patch areas (7.6 ± 2.4 µM), it did not show a significant statistical difference (*t* test: *p* = 0.381) (Fig. 3). On the other hand, the concentrations of DOP showed no significant spatial and temporal changes (Kruskal–Wallis: *p* = 0.494) (Fig. 3). In general, the spatial distributions of higher DON and lower DIN concentrations along the coast during the red tide period of September 17 coincided with the higher peridinin concentration areas (Supplementary Fig. S5).

**Figure 4:**
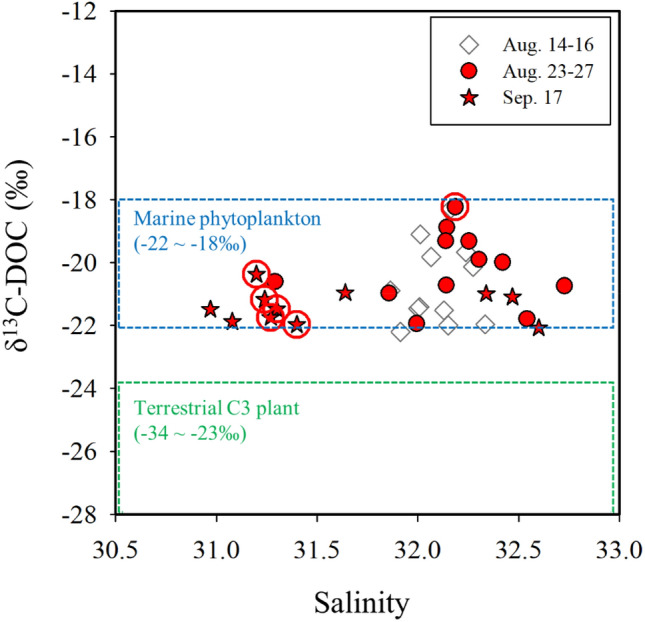
A Plot between δ^13^C-DOC values and salinities in seawaters from the Tongyeong coast during August 14–16, August 23–27, and September 17, 2019. The red circles represent the centers of the red tide patches. The blue dashed box indicates the range of δ^13^C-DOC values of marine phytoplankton-derived DOC, and the green dashed box indicates the range of δ^13^C-DOC values of terrestrial C_3_ plant-derived DOC according to Gearing^24^ and Fry et al.^73^.

The concentrations of DOC during all the sampling periods in 2019 ranged from 71 to 137 µM (average: 90 ± 13 µM) (Fig. 3). In general, higher concentrations of DOC were observed in the low salinity sites before the outbreak of red tides (Supplementary Figs. S1, S2, and S3). However, during the red tide periods, the concentrations of DOC were significantly higher than those before the outbreak of red tides (Kruskal–Wallis: *p* < 0.001) (Fig. 3). The concentrations of DOC in the patch areas (average: 113 ± 14 µM) were 1.2-fold higher than those in the non-patch areas (average: 96 ± 9 µM) during the red tide periods (*t* test: *p* = 0.001). The values of δ^13^C-DOC ranged from − 22.2 to − 18.2‰ for all sampling sites over three periods (before the outbreak: August 14–16, the early stage: August 23–27, and the final stage: September 17) (Fig. 4).

The concentrations of FDOM_C_, FDOM_T_, and FDOM_M_ were in the range of 0.45–2.28 R.U. (average: 0.96 ± 0.43 R.U.), 0.69–2.94 R.U. (average: 1.52 ± 0.54 R.U.), and 0.18–2.12 R.U. (average: 0.89 ± 0.38 R.U.), respectively, for all sampling periods in 2019 (Fig. 3). Similar to the spatial distributions of DOC, the concentrations of FDOM_C_ were generally higher in the low salinity sites before the red tide outbreak (Supplementary Figs. S1, S2, and S3). The concentrations of FDOM_C_ during the red tide periods were significantly higher than those before the outbreak periods (Kruskal–Wallis: *p* < 0.001) (Fig. 3), while FDOM_T_ showed no clear temporal trend (Fig. 3). The concentrations of FDOM_M_ were higher during the red tide periods than those before the red tide outbreak (Kruskal–Wallis: *p* < 0.001) (Fig. 3). During the red tide periods, the average concentrations of FDOM_C_ and FDOM_T_ in the patch areas (average: 1.76 ± 0.04 R.U., 1.96 ± 0.04 R.U., respectively) were 1.7- and 1.3-fold higher than those in the non-patch areas (average: 1.01 ± 0.43 R.U., 1.52 ± 0.23 R.U., respectively) (Mann–Whitney *U* test: *p* < 0.001, *p* = 0.008, respectively), respectively (Fig. 3).

**Figure 5:**
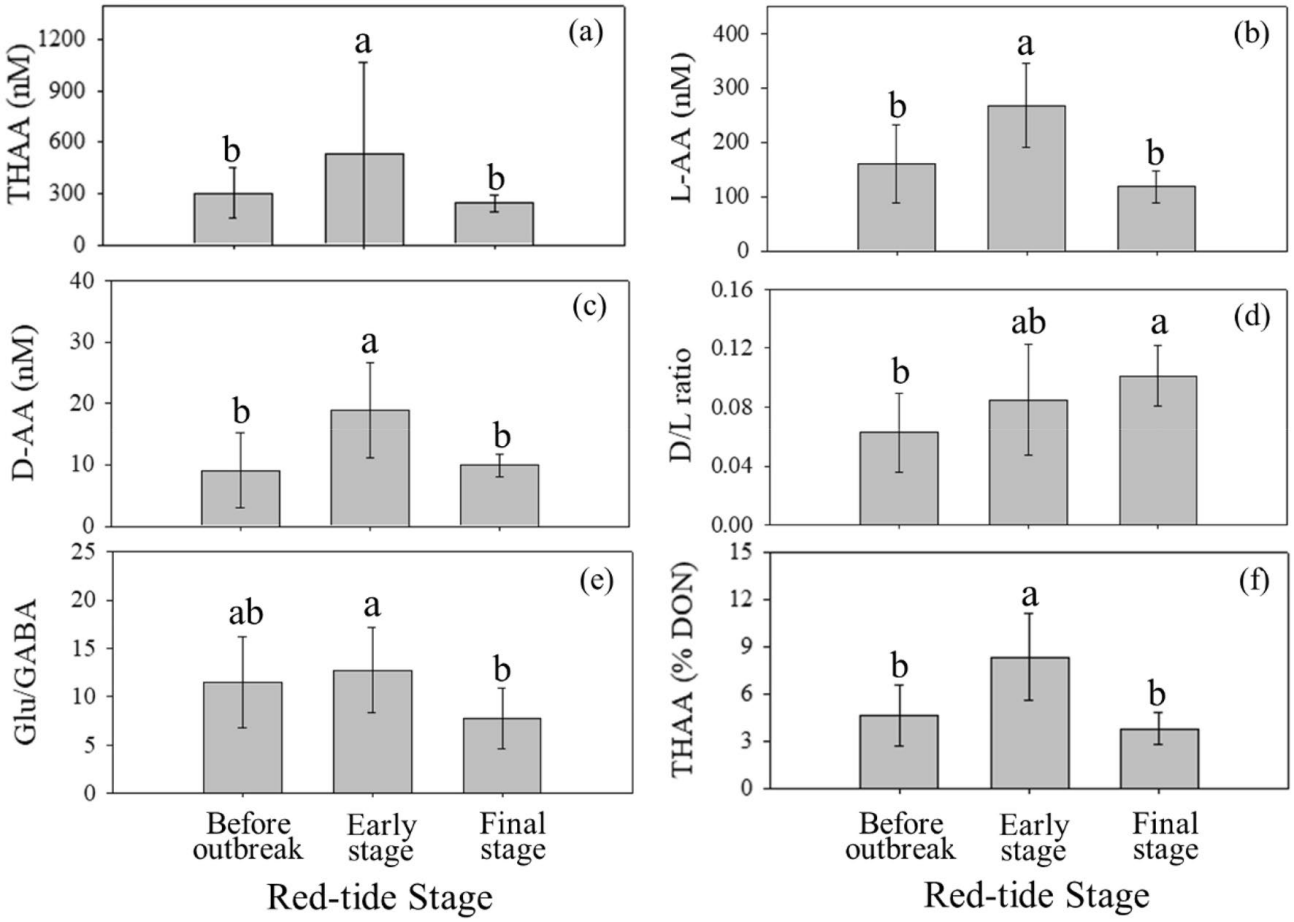
Averages and standard deviations of (**a**) THAA, (**b**) l-AA, (**c**) d-AA, (**d**) D/L ratio, (**e**) Glu/GABA, and (**f**) THAA (%DON) before the outbreak of red tide (August 14–16), the early stage of red tide (August 23–27), and the final stage of red tide (September 17). Significance (*p*) of the differences among the group in all figures was always < 0.05.

The concentrations of THAA, l-AA, and d-AA were in the range of 169–1028 nM, 82–495 nM, and 4–31 nM, respectively, for all sampling sites over the three periods in 2019 (August 14–16, August 23–27, and September 17) (Fig. 5). The average concentrations of these components in the early stage of red tide (average: 536 ± 168 nM for THAA, 268 ± 77 nM for l-AA, and 19 ± 8 nM for d-AA) were about a factor of two higher than those during the other stages (Fig. 5). However, there was no significant difference in the average concentrations of THAA, l-AA, and d-AA between the before‐outbreak period and the final stage period (Fig. 5). The averaged D/L ratios of amino acids ranged from 0.02 to 0.15, which increased gradually according to the red tide stages (one-way ANOVA: *p* = 0.008) (Fig. 5). Except for Ala (Kruskal–Wallis: *p* = 0.647), D/L ratios of individual amino acids (Asp, Glu, and Ser) also increased gradually according to the red tide stages (one-way ANOVA: *p* < 0.001, one-way ANOVA: *p* = 0.007, Kruskal–Wallis: *p* = 0.005, respectively) (Supplementary Table S2). Moreover, there was no significant difference in the D/L ratios of both averaged and individual amino acids between the patch and non-patch areas during the red tide periods (*t*-test for averaged, Asp, and Glu and Mann–Whitney *U* test for Ala and Ser: *p* > 0.05) (Supplementary Table S2). The ratios of Glu/GABA ranged from 3 to 20 for all sampling sites over the three periods (August 14–16, August 23–27, and September 17). The ratios of Glu/GABA were higher in the early stage of red tide (average: 13 ± 4) than those for the other periods (one-way ANOVA: *p* = 0.021) (Fig. 5). However, there was no significant difference in the ratios of Glu/GABA between the patch and non-patch areas during the red tide periods (*t* test: *p* = 0.921) (Supplementary Table S2).

## Discussion

### General characteristics of red tide areas

Peridinin and fucoxanthin are diagnostic indices for dinoflagellates and diatoms, respectively^53^. The concentrations of chl. *a* and peridinin during the red tide period of 2019 were 3.6-fold and 2.1-fold lower than those during the massive red tide outbreak of 2014, respectively^8^ (Fig. 2). However, the concentration of peridinin in 2019 was 6-fold higher than that in 2017 when no red tide outbreaks were observed^8^ (Fig. 2). The concentrations of fucoxanthin showed the highest value during the non-outbreak period^8^ (Fig. 2). This difference in peridinin concentrations seems to be associated with the magnitude of red tides in different years: 2014 (~ 1.5 × 10^4^ cells mL^−1^), 2017 (non-outbreak), and 2019 (~ 5.8 × 10^3^ cells mL^−1^) (http://www.nifs.go.kr/)^40^.

During the red tide periods, except for one station, the concentrations of DIN were depleted (< 2 µM), and DIN/DIP ratios were lower than 11 (Fig. 3), indicating the limitation of biological production by DIN. However, higher DON concentrations were observed during the red tide periods (Fig. 3), consistent with those in previous studies^5,6,8^. In culture experiments, *M. polykrikoides* is able to use various organic nitrogen substances to maintain the growth under the limitation of inorganic nitrogen^7,12^. Although *M. polykrikoides* can utilize inorganic nutrients in bottom waters through vertical migration^13^, the concentrations of DIN in the bottom waters during the red tide period of September 17 were < 2.5 μM (average: 1.2 ± 0.8 µM, data are not shown). This means that, in addition to vertical migration, the utilization of organic nutrients is critical for the persistence of red tides. Thus, the areal distributions of DIN and DON during the red tide outbreak periods seem to be similar to the red tide distribution patterns (Supplementary Figs. S4 and S5).

### Origins of DOC and FDOM in red tide areas

**Figure 6:**
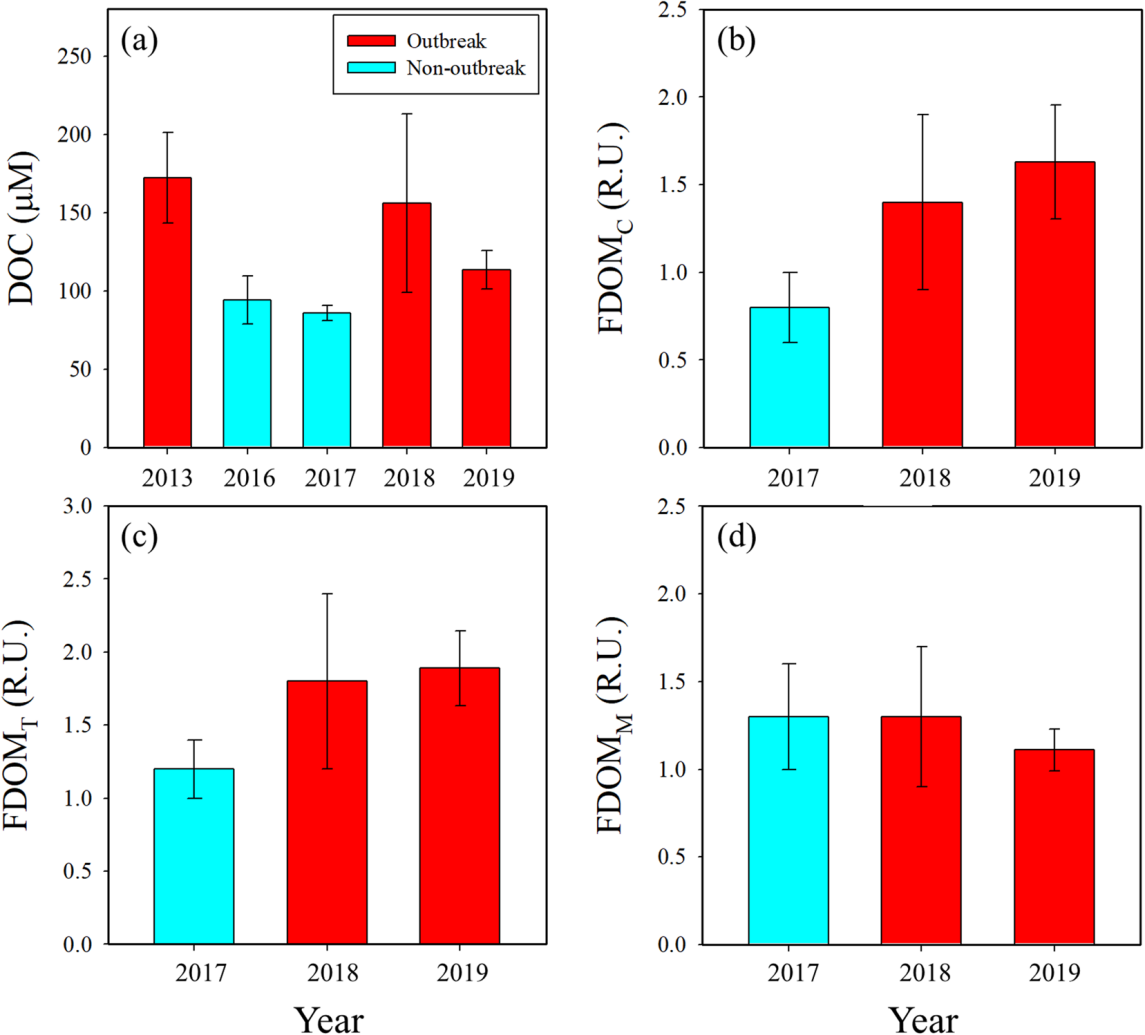
Average concentrations of (**a**) DOC, (**b**) FDOM_C_, (**c**) FDOM_T_, and (**d**) FDOM_M_ in coastal waters off Tongyeong, Korea, during the summers from 2013 to 2019. The DOC concentrations in 2013 and 2016 in the red tide regions of Tongyeong were obtained from Kwon et al.^14^.

In river-dominated coastal oceans, the distribution of DOC is generally dependent on salinity^54,55^. However, in this study region, the distribution of DOC concentrations was independent of the salinity, with enhanced DOC concentrations in the patch areas (Fig. 3). This trend is consistent with the previous observations in this region from different years (i.e., from 2013 to 2018) (Fig. 6). A previous culture experiment showed that *M. polykrikoides* significantly release various organic substances, such as polysaccharides, proteins, amino acids, and carbohydrates^56^. Thus, the concentrations of DOC were higher in the patch areas than in the non-patch areas (Fig. 3). The values of δ^13^C-DOC (ranged from − 22.2 to − 18.2‰) fall into the range of marine sources of DOC (ranged from − 22 to − 18‰), indicating that the main source of DOC in the red tide areas is a marine origin (Fig. 4). This result is consistent with the previous hypothesis^8^ that DOM fueling red tides in this region is produced mainly by microbial production following the massive diatom growth^57,58^ and abrupt cessation of DIN supply.

In general, the major source of FDOM_C_ in coastal waters is known to originate mainly from terrestrial freshwater, as shown by significant negative correlations between FDOM_C_ and salinity^59,60^. As such, in Gwangyang Bay, which is located between Yeosu and Namhae, Korea, FDOM_C_ concentrations showed significant negative correlations (r^2^ = 0.92, *p* < 0.0001) against salinity^61^. However, the distribution of FDOM_C_ in this study region was independent of the salinity, similar to that of DOC (Fig. 3). Our observations show that the concentrations of both FDOM_C_ and FDOM_T_ were higher in the patch areas (Fig. 3). Culture experiments have shown that FDOM_C_ can be produced directly from phytoplankton and microbial transformation of planktonic materials^62^. Also, FDOM_T_ is known to be produced by biological activities^63,64^. Previous field observations in the same region also showed the relatively enhanced concentrations of FDOM_C_ and FDOM_T_ during the red tide periods compared with those for the non-outbreak periods (Fig. 6). Thus, it is likely that FDOM_C_ and FDOM_T_ are produced in the red tide region as suggested by Kwon et al.^14^.

In contrast, there was no significant difference in the concentrations of FDOM_M_ between the patch and non-patch areas (Fig. 3), though the distribution pattern of FDOM_M_ was generally similar to that of FDOM_C_^22,48,65^. FDOM_M_ can be produced either by bacterial activities^65^ or phytoplankton^63^ and also can be taken up by bacteria^66^. In general, the consumption rates of FDOM_M_ by bacteria are approximately 2-fold higher than the production rates by phytoplankton^66^. Thus, the preferential consumption of FDOM_M_ by bacteria in red tide areas where higher production occurs could result in similar concentrations in both red tide periods and non-red tide periods (Fig. 6), as previously shown by Kwon et al.^14^.

### Characteristics of DOM revealed by amino-acid indices

Most of the amino acids have enantiomeric forms with l- and d-enantiomers in seawater^67^. While the proteins are composed of l-enantiomers in most organisms, d-enantiomers are known to be mostly derived from bacterial cell membranes. In general, d-enantiomers tend to be accumulated during DOM degradation because of the key role of bacteria in DOM degradation^68,69^. A higher enantiomeric (D/L) ratio of amino acids, therefore, indicates that DOM is more refractory. Similarly, the ratio of glutamic acid to GABA (Glu/GABA) is one of the biodegradation indices since GABA, a non-protein amino acid, is accumulated by the degradation of glutamic acid during microbial degradation^30^. As mentioned above, the averaged D/L ratios of amino acids increased gradually according to the red tide stages in the study region (one-way ANOVA: *p* = 0.008) (Fig. 5). The ratios of Glu/GABA were also higher in the early stage of red tide (average: 13 ± 4) than those for the other periods (one-way ANOVA: *p* = 0.021) (Fig. 5). Thus, it is likely that fresh amino acids decrease in the course of the red tide succession.

THAA (%DON) has been widely used for characterizing the bioavailability of DOM^70–72^. Our results show that the yields of THAA (%DON) in the early stage of red tide (average: 8 ± 3%) were significantly higher than those for the other periods (Fig. 5). This trend consistently indicates that bioavailable DON concentrations were highest in the early stage of red tide and diminished in the final stage (Fig. 5). Therefore, all biogeochemical parameters (D/L ratio, Glu/GABA, and THAA [%DON]) suggest that the amount of bioavailable DOM is critical for the occurrence of red tides if other conditions (i.e., depleted inorganic nutrients, temperature, and salinity) are favorable for red tide outbreaks.

## Conclusions

The environmental conditions of *M. polykrikoides* red tide occurrence in the coastal region off Tongyeong show depleted concentrations of inorganic nitrogen and enhanced concentrations of organic components (DON, DOC, FDOM, and THAA). These conditions are favorable for the growth of dinoflagellates in competition with diatoms. Our results show that the enhanced DOC is produced in-situ by biological activities based on the δ^13^C-DOC values (− 22.2 to − 18.2‰). In addition, we found that bioavailable DOM increased in the early stage of red tide and decreased in the final stage, based on various degradation indices (D/L ratio, Glu/GABA, and THAA [%DON]). Our results reveal that freshly produced DOM plays an important role in the red tide outbreak, together with other environmental conditions. Therefore, our tools for determining the sources and origins of DOM in this red tide region can be utilized similarly in other red tide areas of the global ocean.

## Supplementary Information

Supplementary Information.

## Data Availability

The datasets analysed during the current study are available from the corresponding author on reasonable request.
