# Supplementary material for: Origins and characteristics of dissolved organic matter fueling harmful dinoflagellate blooms revealed by δ13C and d/l-Amino acid compositions
Source: Sci Rep. 2022 Sep 5;12:15052. doi: 10.1038/s41598-022-19168-7 (PMC9445177; doi:10.1038/s41598-022-19168-7)
Supplement: Supplementary file 1 — Supplementary Information. [file 41598_2022_19168_MOESM1_ESM.docx]

**Supplementary Information**

**Origins and characteristics of dissolved organic matter fueling harmful dinoflagellate blooms revealed by δ^13^C and D/L-Amino acid compositions**

Jihyun Park^1^, Guebuem Kim^1*^, Hyeong Kyu Kwon^1^, Heejun Han^1^, Tae Gyu Park^2^, Moonho Son^3^

^1^School of Earth and Environmental Sciences/Research Institute of Oceanography, Seoul National University, Seoul, 08826, Republic of Korea

^2^Southeast Sea Fisheries Research Institute, National Institute of Fisheries Science, Tongyeong, 53085, Republic of Korea

^3^Ocean Climate and Ecology Research Division, National Institute of Fisheries Science, Busan, 46083, Republic of Korea

^*^Corresponding author: Guebuem Kim (gkim@snu.ac.kr)

**
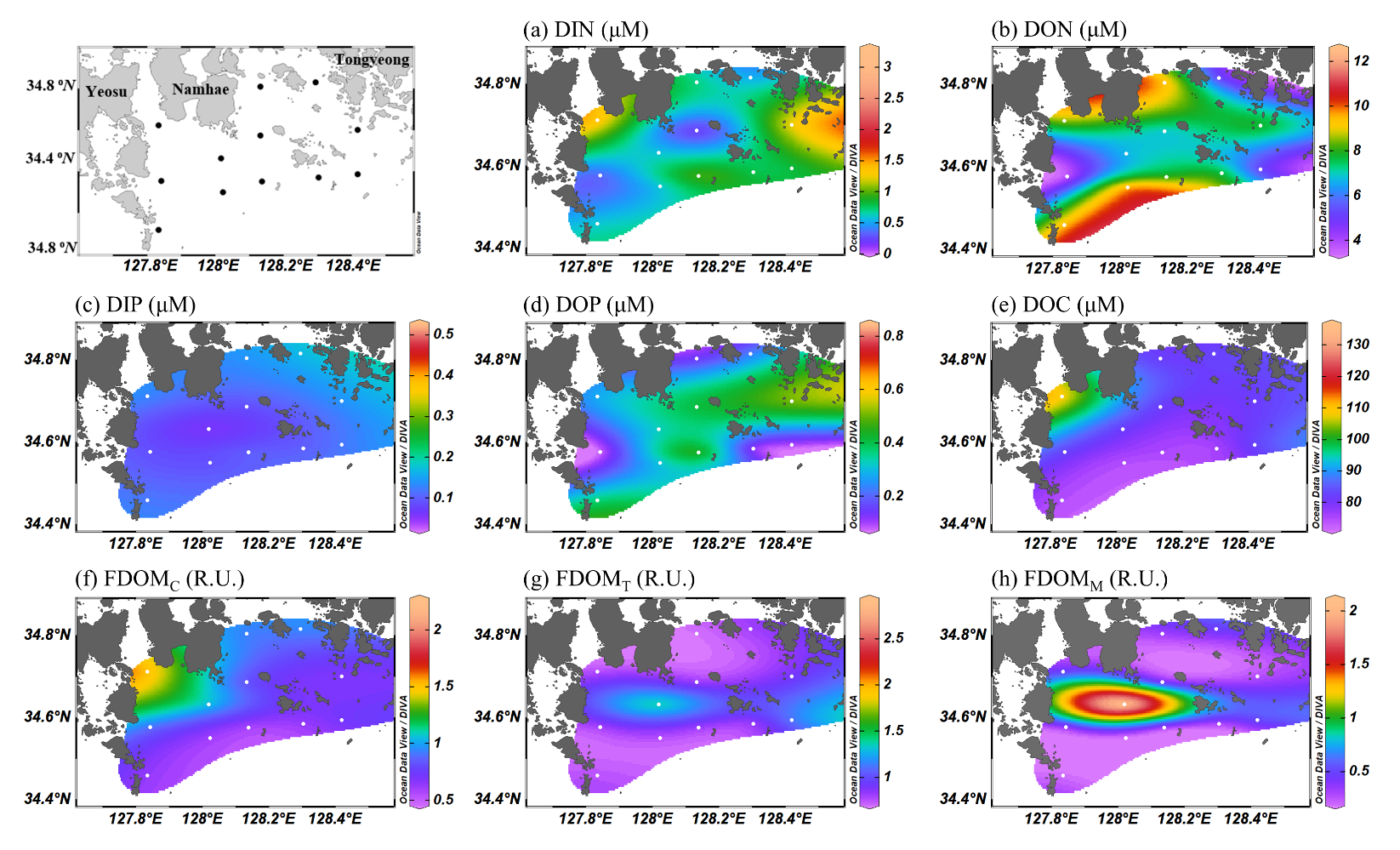
**

Supplementary Figure S1. Map of the sampling stations and distributions of (a) DIN, (b) DON, (c) DIP, (d) DOP, (e) DOC, (f) FDOM_C_, (g) FDOM_T_, and (h) FDOM_M_ in seawaters off Tongyeong during May 30–June 16, 2019 (before the outbreak of red tides). The contour plots were produced by Ocean Data View (https://odv.awi.de).

**
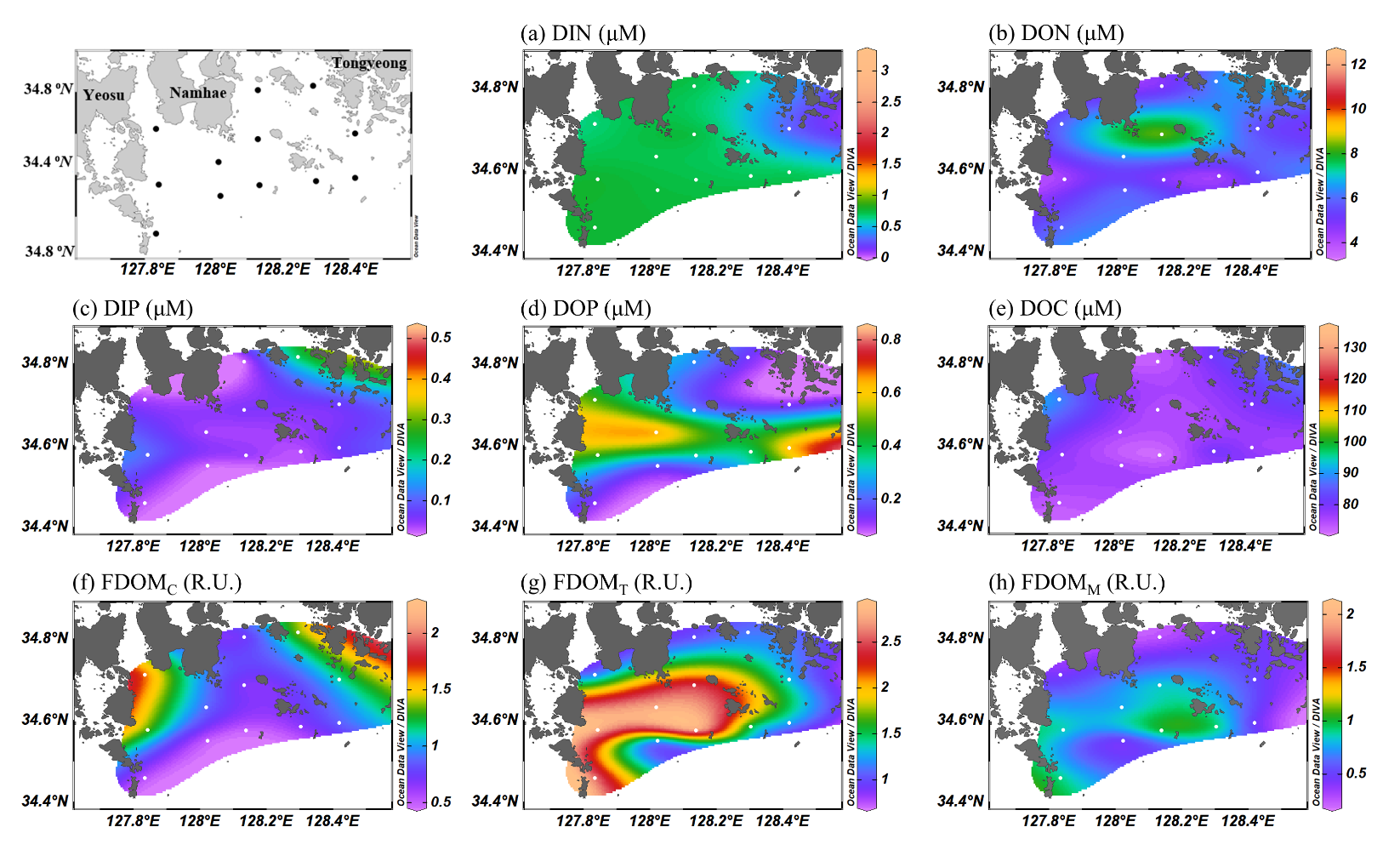
**

Supplementary Figure S2. Map of the sampling stations and distributions of (a) DIN, (b) DON, (c) DIP, (d) DOP, (e) DOC, (f) FDOM_C_, (g) FDOM_T_, and (h) FDOM_M_ in seawaters off Tongyeong during July 17–19, 2019 (before the outbreak of red tides). The contour plots were produced by Ocean Data View (https://odv.awi.de).


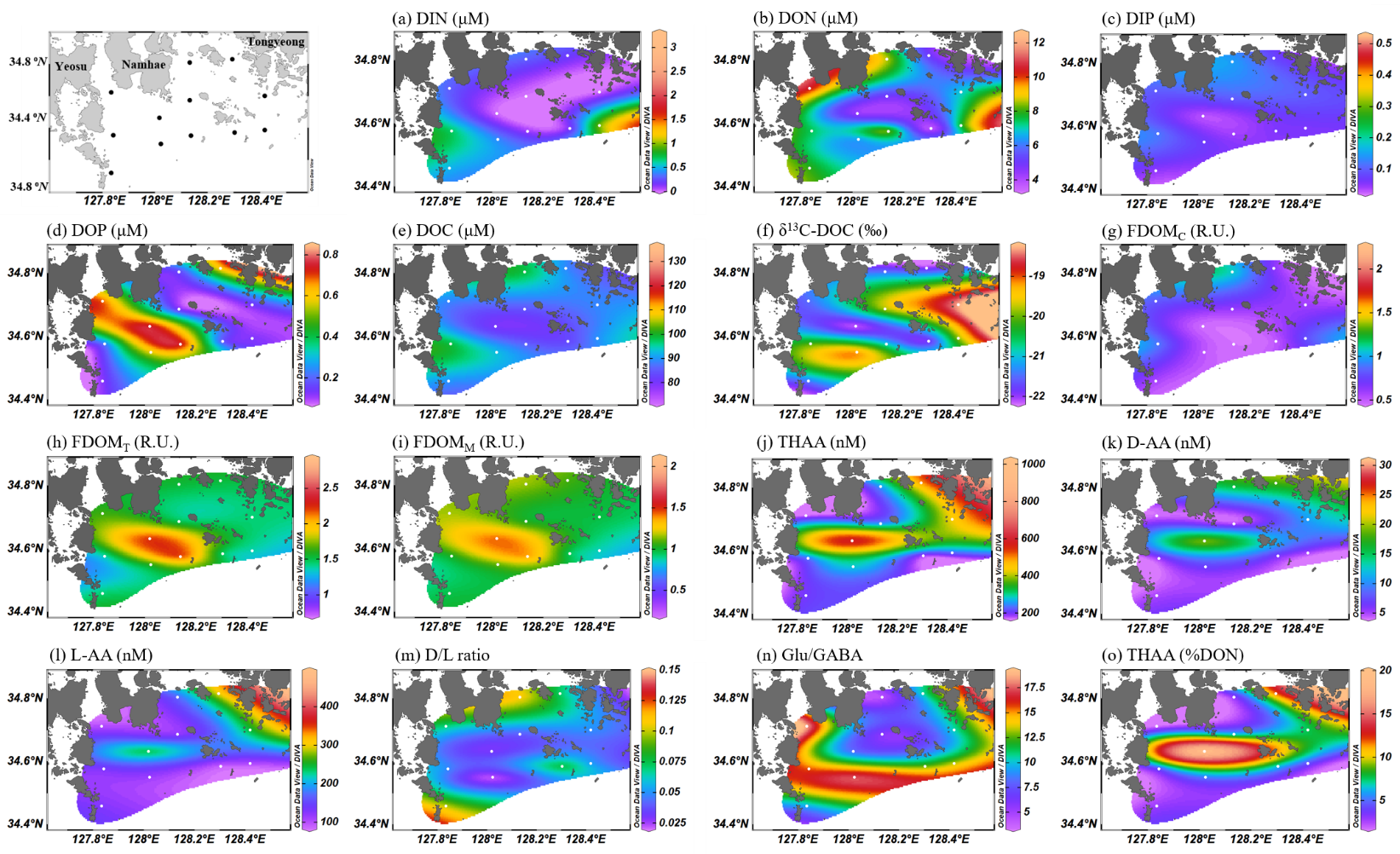


Supplementary Figure S3. Map of the sampling stations and distributions of (a) DIN, (b) DON, (c) DIP, (d) DOP, (e) DOC, (f) δ^13^C-DOC, (g) FDOM_C_, (h) FDOM_T_, (i) FDOM_M_, (j)THAA, (k) D-AA, (l) L-AA, (m) D/L ratio, (n) Glu/GABA, and (o) THAA (%DON) in seawaters off Tongyeong during August 14–16, 2019 (before the outbreak of red tides). The contour plots were produced by Ocean Data View (https://odv.awi.de).


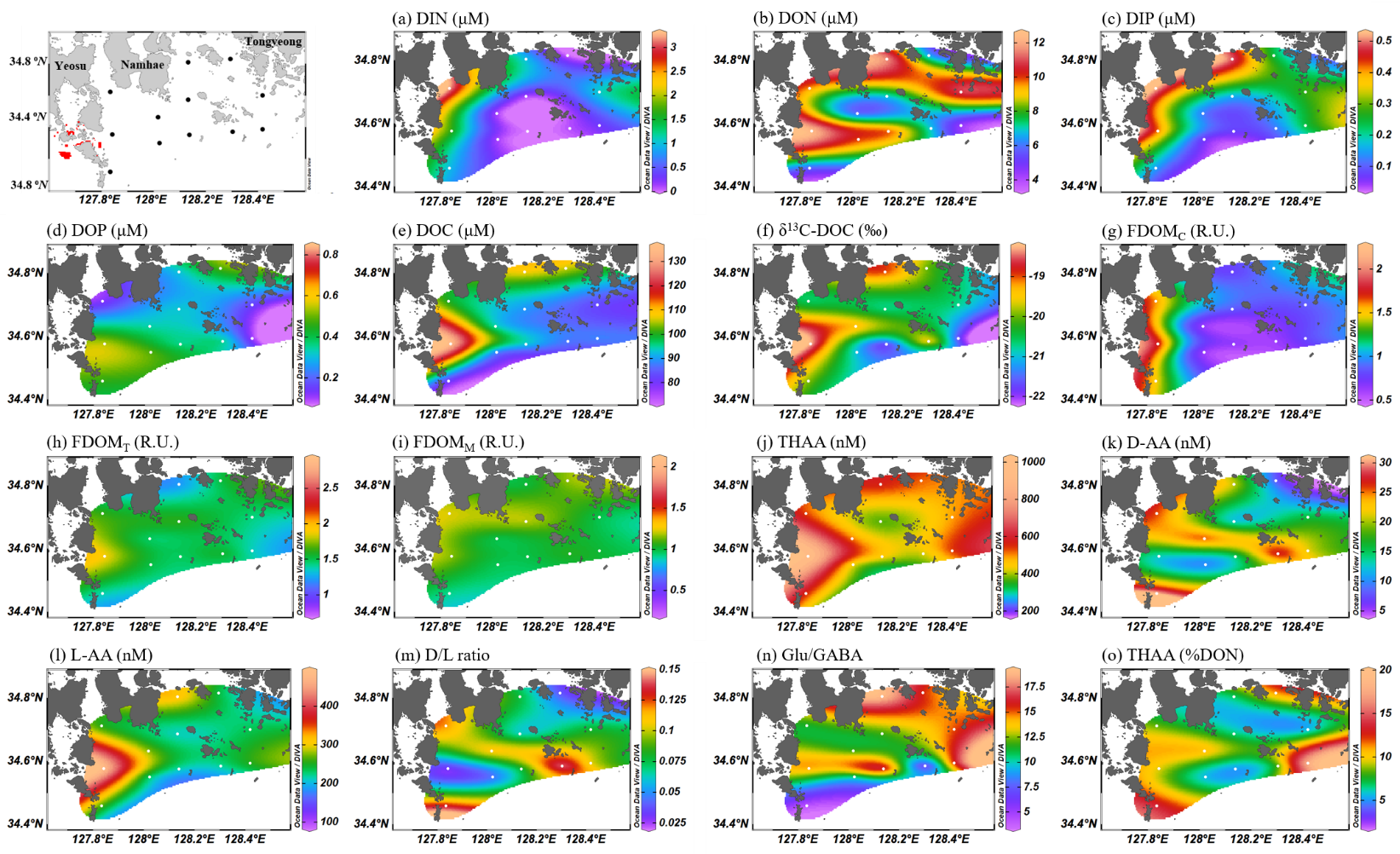


Supplementary Figure S4. Map of the sampling stations and distributions of (a) DIN, (b) DON, (c) DIP, (d) DOP, (e) DOC, (f) δ^13^C-DOC, (g) FDOM_C_, (h) FDOM_T_, (i) FDOM_M_, (j)THAA, (k) D-AA, (l) L-AA, (m) D/L ratio, (n) Glu/GABA, and (o) THAA (%DON) in seawaters off Tongyeong during August 23–27, 2019 (the early stage of red tides). The contour plots were produced by Ocean Data View (https://odv.awi.de).


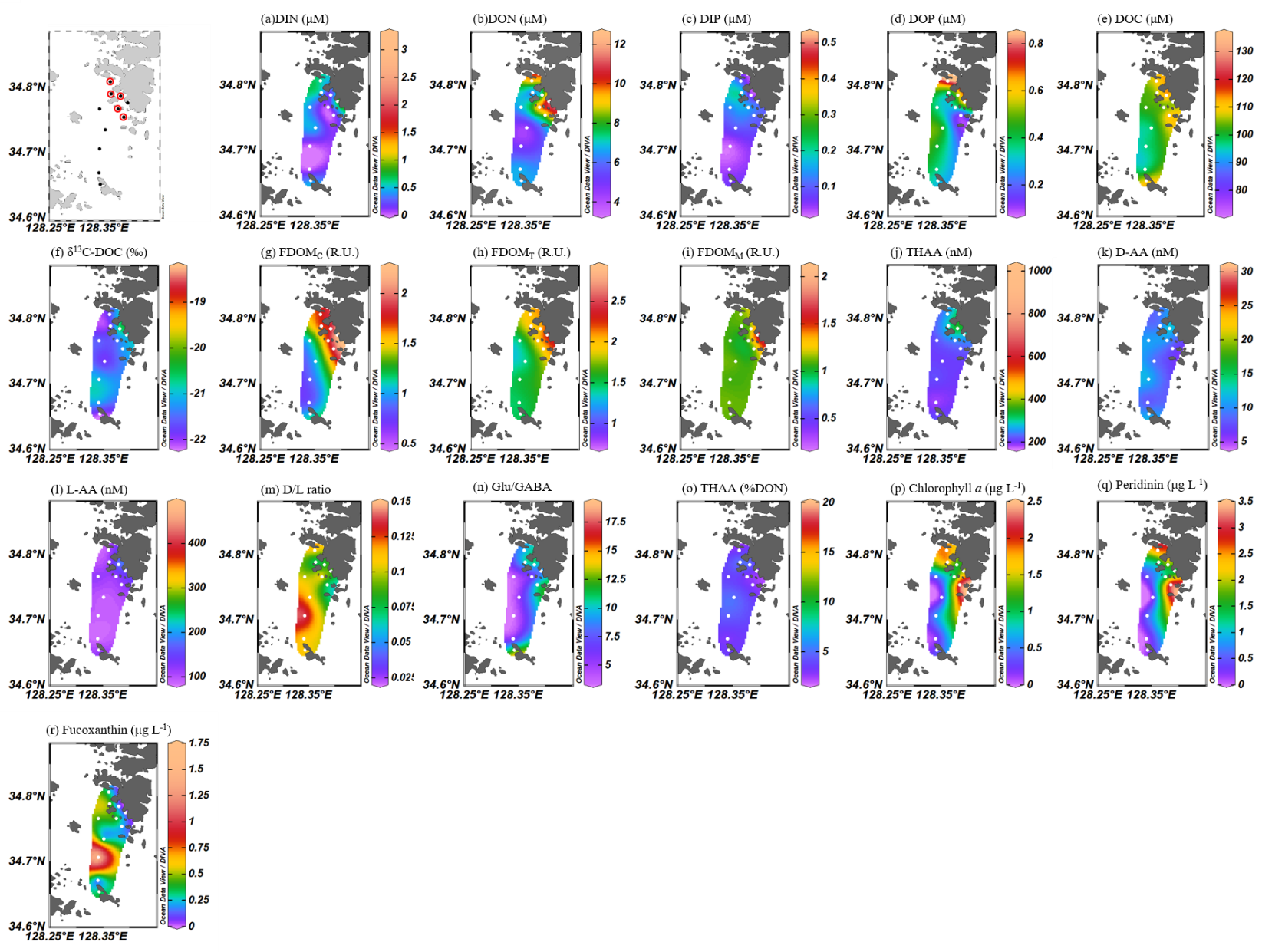


Supplementary Figure S5. Distributions of (a) DIN, (b) DON, (c) DIP, (d) DOP, (e) DOC, (f) δ^13^C-DOC, (g) FDOM_C_, (h) FDOM_T_, (i) FDOM_M_, (j)THAA, (k) D-AA, (l) L-AA, (m) D/L ratio, (n) Glu/GABA, (o) THAA (%DON), (p) chlorophyll *a*, (q) peridinin, and (r) fucoxanthin in the coastal region off Tongyeong on September 17, 2019 (the final stage of red tides). The contour plots were produced by Ocean Data View (https://odv.awi.de).

**Table S1.** Salinity and the concentrations of DIN, DON, DIP, DOP, FDOM_C_, FDOM_T_, FDOM_M_, and DOC in seawaters off Tongyeong, Korea during the summers of 2017, 2018, and 2019.

| **Station** | | | | | **Salinity** | **Nutrients (μM)** | | | | **FDOM (R.U.)** | | | **DOC** |
| --- | --- | --- | --- | --- | --- | --- | --- | --- | --- | --- | --- | --- | --- |
|  | **Type** | **St.** | **Longitude** | **Latitude** |  | **DIN** | **DON** | **DIP** | **DOP** | **FDOM_C_** | **FDOM_T_** | **FDOM_M_** |  |
| May 30-Jun.5, 2019 | Non-patch area | T1 | 128.418 | 34.701 | 33.79 | 1.3 | 7.8 | 0.14 | 0.51 | 0.70 | 0.91 | 0.28 | 82 |
|  |  | T2 | 128.418 | 34.5939 | 33.75 | 0.8 | 5.3 | 0.12 | 0.10 | 0.72 | 1.13 | 0.53 | 83 |
|  |  | T3 | 128.294 | 34.8162 | 33.60 | 0.5 | 5.2 | 0.17 | 0.23 | 0.95 | 0.81 | 0.48 | 83 |
|  |  | T4 | 128.303 | 34.5857 | 33.86 | 0.8 | 7.8 | 0.10 | 0.10 | 0.64 | 0.82 | 0.31 | 76 |
|  |  | T5 | 128.133 | 34.8052 | 33.48 | 0.7 | 9.5 | 0.14 | 0.14 | 0.93 | 0.69 | 0.35 | 82 |
|  |  | T6 | 128.133 | 34.6872 | 33.44 | 0.2 | 6.9 | 0.09 | 0.41 | 0.82 | 0.81 | 0.39 | 82 |
|  |  | T7 | 128.137 | 34.5765 | 33.77 | 0.1 | 7.9 | 0.10 | 0.46 | 0.60 | 0.82 | 0.32 | 76 |
|  |  | T8 | 128.017 | 34.6324 | 33.54 | 0.6 | 6.8 | 0.06 | 0.33 | 1.06 | 1.38 | 2.12 | 82 |
|  |  | T9 | 128.023 | 34.5500 | 33.79 | 0.6 | 9.8 | 0.09 | 0.31 | 0.62 | 0.77 | 0.18 | 75 |
|  |  | T10 | 127.833 | 34.7119 | 32.61 | 1.4 | 8.6 | 0.13 | 0.28 | 1.53 | 0.77 | 0.49 | 109 |
|  |  | T11 | 127.833 | 34.4595 | 33.72 | 0.6 | 9.4 | 0.12 | 0.37 | 0.65 | 0.76 | 0.20 | 75 |
|  |  | T12 | 127.842 | 34.5775 | 33.34 | 0.4 | 3.7 | 0.09 | 0.07 | 0.91 | 0.80 | 0.36 | 83 |
| Jul.17-19, 2019 | Non-patch area | T1 | 128.418 | 34.7010 | 33.07 | 0.3 | 5.6 | 0.07 | 0.12 | 1.13 | 1.30 | 0.53 | 80 |
|  |  | T2 | 128.418 | 34.5939 | 33.05 | 0.7 | 5.8 | 0.07 | 0.63 | 0.86 | 1.28 | 0.48 | 77 |
|  |  | T3 | 128.294 | 34.8162 | 32.79 | 0.6 | 6.4 | 0.23 | 0.14 | 1.38 | 1.13 | 0.40 | 81 |
|  |  | T4 | 128.303 | 34.5857 | 33.03 | 0.7 | 4.4 | 0.03 | 0.37 | 0.67 | 1.46 | 0.94 | 81 |
|  |  | T5 | 128.133 | 34.8052 | 32.35 | 0.7 | 4.8 | 0.02 | 0.25 | 0.73 | 1.01 | 0.27 | 73 |
|  |  | T6 | 128.133 | 34.6872 | 32.51 | 0.8 | 8.4 | 0.06 | 0.23 | 0.68 | 2.45 | 0.84 | 75 |
|  |  | T7 | 128.137 | 34.5765 | 32.91 | 0.7 | 4.8 | 0.05 | 0.35 | 0.50 | 2.94 | 1.02 | 71 |
|  |  | T8 | 128.017 | 34.6324 | 32.66 | 0.7 | 6.4 | 0.04 | 0.66 | 0.89 | 2.78 | 0.76 | 76 |
|  |  | T9 | 128.023 | 34.5500 | 32.89 | 0.8 | 6.0 | 0.02 | 0.17 | 0.50 | 1.12 | 0.45 | 76 |
|  |  | T10 | 127.833 | 34.7119 | 32.29 | 0.7 | 5.9 | 0.04 | 0.48 | 1.66 | 1.14 | 0.44 | 89 |
|  |  | T11 | 127.833 | 34.4595 | 32.87 | 0.8 | 6.0 | 0.03 | 0.12 | 0.56 | 2.63 | 0.91 | 74 |
|  |  | T12 | 127.842 | 34.5775 | 32.93 | 0.8 | 4.4 | 0.11 | 0.47 | 1.34 | 2.87 | 0.87 | 78 |
| Aug. 14-16, 2019 | Non-patch area | T1 | 128.418 | 34.7010 | 32.17 | 0.0 | 6.9 | 0.10 | 0.14 | 0.60 | 1.45 | 0.98 | 89 |
|  |  | T2 | 128.418 | 34.5939 | 32.07 | 1.0 | 8.1 | 0.06 | 0.14 | 0.80 | 1.39 | 0.87 | 90 |
|  |  | T3 | 128.294 | 34.8162 | 32.34 | 0.2 | 5.0 | 0.08 | 0.63 | 0.64 | 1.53 | 1.08 | 89 |
|  |  | T4 | 128.303 | 34.5857 | 32.15 | 0.1 | 3.3 | 0.06 | 0.15 | 0.51 | 1.43 | 0.93 | 83 |
|  |  | T5 | 128.133 | 34.8052 | 32.13 | 0.3 | 8.7 | 0.15 | 0.15 | 1.09 | 1.54 | 1.16 | 99 |
|  |  | T6 | 128.133 | 34.6872 | 32.24 | 0.0 | 5.0 | 0.10 | 0.11 | 0.63 | 1.47 | 1.02 | 85 |
|  |  | T7 | 128.137 | 34.5765 | 31.86 | 0.1 | 8.8 | 0.04 | 0.78 | 0.45 | 2.26 | 1.40 | 86 |
|  |  | T8 | 128.017 | 34.6324 | 31.92 | 0.0 | 4.3 | 0.03 | 0.77 | 0.46 | 2.25 | 1.44 | 80 |
|  |  | T9 | 128.023 | 34.5500 | 32.01 | 0.4 | 6.4 | 0.07 | 0.57 | 0.52 | 1.59 | 1.10 | 93 |
|  |  | T10 | 127.833 | 34.7119 | 32.00 | 0.3 | 10.5 | 0.10 | 0.70 | 0.83 | 1.65 | 1.17 | 89 |
|  |  | T11 | 127.833 | 34.4595 | 32.01 | 0.5 | 8.0 | 0.05 | 0.12 | 0.58 | 1.45 | 0.97 | 90 |
|  |  | T12 | 127.842 | 34.5775 | 32.27 | 0.7 | 7.5 | 0.12 | 0.21 | 0.91 | 1.36 | 1.02 | 99 |
| Aug.23-27, 2019 | Non-patch area | T1 | 128.418 | 34.7010 | 32.73 | 1.1 | 11.0 | 0.28 | 0.16 | 0.86 | 1.44 | 0.98 | 83 |
|  |  | T2 | 128.418 | 34.5939 | 32.54 | 0.3 | 4.4 | 0.24 | 0.10 | 0.81 | 1.36 | 1.01 | 88 |
|  |  | T3 | 128.294 | 34.8162 | 32.42 | 0.0 | 6.4 | 0.24 | 0.49 | 1.03 | 1.53 | 1.17 | 106 |
|  |  | T4 | 128.303 | 34.5857 | 32.25 | 0.1 | 6.9 | 0.12 | 0.37 | 0.62 | 1.57 | 1.05 | 87 |
|  |  | T5 | 128.133 | 34.8052 | 32.14 | 1.0 | 11.8 | 0.50 | 0.31 | 0.78 | 1.21 | 1.00 | 108 |
|  |  | T6 | 128.133 | 34.6872 | 32.14 | 0.1 | 6.9 | 0.16 | 0.32 | 0.87 | 1.62 | 1.20 | 90 |
|  |  | T7 | 128.137 | 34.5765 | 31.99 | 0.0 | 10.0 | 0.08 | 0.35 | 0.68 | 1.47 | 0.99 | 94 |
|  |  | T8 | 128.017 | 34.6324 | 32.14 | 0.1 | 5.7 | 0.12 | 0.34 | 0.62 | 1.46 | 0.98 | 94 |
|  |  | T9 | 128.023 | 34.5500 | 31.86 | 0.1 | 9.9 | 0.06 | 0.49 | 0.60 | 1.51 | 0.97 | 87 |
|  |  | T10 | 127.833 | 34.7119 | 31.29 | 3.3 | 9.6 | 0.53 | 0.12 | 1.61 | 1.63 | 1.23 | 100 |
|  |  | T11 | 127.833 | 34.4595 | 32.30 | 1.0 | 5.8 | 0.15 | 0.46 | 1.49 | 1.39 | 0.92 | 82 |
|  | Patch area | T12 | 127.842 | 34.5775 | 32.19 | 1.7 | 12.6 | 0.43 | 0.55 | 1.78 | 1.90 | 1.16 | 137 |

**Table S1.** Continued.

| **Station** | | | | | **Salinity** | **Nutrients (μM)** | | | | **FDOM (R.U.)** | | | **DOC** |
| --- | --- | --- | --- | --- | --- | --- | --- | --- | --- | --- | --- | --- | --- |
|  | **Type** | **St.** | **Longitude** | **Latitude** |  | **DIN** | **DON** | **DIP** | **DOP** | **FDOM_C_** | **FDOM_T_** | **FDOM_M_** |  |
| Sep.19, 2019 | Non-patch area | S1 | 128.343 | 34.6535 | 32.60 | 0.4 | 6.6 | 0.06 | 0.31 | 1.07 | 1.49 | 1.11 | 109 |
|  |  | S2 | 128.34 | 34.6709 | 32.47 | 0.0 | 6.8 | 0.04 | 0.37 | 0.79 | 1.47 | 1.13 | 96 |
|  |  | S3 | 128.341 | 34.7067 | 32.34 | 0.0 | 4.9 | 0.02 | 0.43 | 0.86 | 1.54 | 1.08 | 97 |
|  |  | S4 | 128.352 | 34.7350 | 31.08 | 0.5 | 4.4 | 0.10 | 0.46 | 1.04 | 1.41 | 1.12 | 97 |
|  |  | S5 | 128.341 | 34.7663 | 30.97 | 0.4 | 6.9 | 0.13 | 0.36 | 1.09 | 1.44 | 1.07 | 103 |
|  |  | S6 | 128.392 | 34.7753 | 31.64 | 0.9 | 11.2 | 0.11 | 0.67 | 2.28 | 2.32 | 1.66 | 104 |
|  | Patch area | S7 | 128.375 | 34.7663 | 31.30 | 0.0 | 9.9 | 0.16 | 0.35 | 1.71 | 1.94 | 1.07 | 103 |
|  |  | S8 | 128.362 | 34.7890 | 31.40 | 0.6 | 6.3 | 0.21 | 0.36 | 1.75 | 2.00 | 1.12 | 105 |
|  |  | S9 | 128.385 | 34.7539 | 31.24 | 0.0 | 8.2 | 0.11 | 0.09 | 1.84 | 1.94 | 1.08 | 111 |
|  |  | S10 | 128.361 | 34.8070 | 31.27 | 0.6 | 9.5 | 0.10 | 0.85 | 1.74 | 2.00 | 1.13 | 109 |
|  |  | S11 | 128.38 | 34.7853 | 31.20 | 0.0 | 7.0 | 0.02 | 0.50 | 1.73 | 1.98 | 1.08 | 115 |
| Jul.30-Aug.3, 2018 | Patch area | T1 | 128.417 | 34.701 | 32.13 | - | - | - | - | 2.01 | 1.75 | 1.66 | 150 |
|  |  | T3 | 128.294 | 34.8162 | 31.88 | - | - | - | - | 1.7 | 2.07 | 1.58 | 151 |
|  |  | T5 | 128.132 | 34.8052 | 31.75 | - | - | - | - | 1.69 | 2.1 | 1.6 | 154 |
|  |  | T8 | 128.017 | 34.6324 | 31.85 | - | - | - | - | 0.75 | 0.96 | 0.86 | 97 |
|  |  | T9 | 128.022 | 34.55 | 31.75 | - | - | - | - | 0.65 | 1.03 | 0.95 | 99 |
|  |  | T10 | 127.833 | 34.7119 | 31.47 | - | - | - | - | 1.45 | 2.4 | 1.54 | 171 |
|  |  | T11 | 127.833 | 34.4595 | 32.1 | - | - | - | - | 1.98 | 2.6 | 1.57 | 267 |
|  | Non-patch area | T2 | 128.417 | 34.5939 | 31.9 | - | - | - | - | 0.52 | 1.49 | 0.73 | 96 |
|  |  | T4 | 128.302 | 34.5857 | 31.88 | - | - | - | - | 0.83 | 0.93 | 0.6 | 95 |
|  |  | T6 | 128.132 | 34.6872 | 32.01 | - | - | - | - | 0.99 | 1.7 | 1.09 | 97 |
|  |  | T7 | 128.137 | 34.5765 | 30.11 | - | - | - | - | 0.72 | 1.13 | 0.72 | 77 |
|  |  | T12 | 127.841 | 34.5775 | 31.8 | - | - | - | - | 1.13 | 1.81 | 1.4 | 89 |
| Jul.31-Aug.1, 2017 | Non-patch area | N1 | 128.418 | 34.7011 | 33.09 | - | - | - | - | 0.94 | 0.98 | 1.5 | 80 |
|  |  | N2 | 128.414 | 34.5135 | 33.48 | - | - | - | - | 0.85 | 1.11 | 1.32 | 78 |
|  |  | N3 | 128.292 | 34.8152 | 33.79 | - | - | - | - | 0.92 | 1.2 | 1.47 | 82 |
|  |  | N4 | 128.27 | 34.5917 | 32.28 | - | - | - | - | 0.79 | 1.5 | 1.18 | 88 |
|  |  | N5 | 128.135 | 34.8104 | 33.38 | - | - | - | - | 1.15 | 0.92 | 1.86 | 83 |
|  |  | N6 | 128.12 | 34.6972 | 32.12 | - | - | - | - | 0.79 | 1.62 | 1.28 | 95 |
|  |  | N7 | 128.123 | 34.5342 | 31.6 | - | - | - | - | 0.7 | 1.34 | 1.15 | 89 |
|  |  | N8 | 128.018 | 34.6526 | 32.18 | - | - | - | - | 0.76 | 1.13 | 1.1 | 89 |
|  |  | N9 | 128.009 | 34.4763 | 31.48 | - | - | - | - | 0.69 | 1.4 | 1.06 | 92 |
|  |  | N10 | 127.828 | 34.6961 | 33.44 | - | - | - | - | 1.14 | 1.48 | 1.71 | 89 |
|  |  | N11 | 127.825 | 34.5724 | 32.16 | - | - | - | - | 0.78 | 0.94 | 1.16 | 83 |
|  |  | N12 | 127.842 | 34.4949 | 31.7 | - | - | - | - | 0.66 | 1.31 | 1.04 | 86 |

**Table S2.** The δ^13^C-DOC values and the concentrations of Asp, Ala, Glu, Ser, Thr, Gly, Arg, Tyr, Val, Phe, Leu, Ile, and GABA in seawaters off Tongyeong, Korea from August to September 2019.

| **Station** | | | | | **δ^13^C-DOC (‰)** | **Amino acids (nM)** | | | | | | | | | | | | | | | | |
| --- | --- | --- | --- | --- | --- | --- | --- | --- | --- | --- | --- | --- | --- | --- | --- | --- | --- | --- | --- | --- | --- | --- |
|  |  |  |  |  |  | **D-** | | | | **L-** | | | | **Thr** | **Gly** | **Arg** | **Tyr** | **Val** | **Phe** | **Leu** | **Ile** | **GABA** |
|  | **Type** | **St.** | **Longitude** | **Latitude** |  | **Asp** | **Ala** | **Glu** | **Ser** | **Asp** | **Ala** | **Glu** | **Ser** |  |  |  |  |  |  |  |  |  |
| Aug. 14-16, 2019 | Non-patch area | T1 | 128.418 | 34.7010 | -18.3 | 5 | 4 | 2 | 0 | 59 | 74 | 61 | 56 | 30 | 117 | 30 | 0 | 1 | 0 | 1 | 0 | 6 |
|  |  | T2 | 128.418 | 34.5939 | -19.8 | 2 | 1 | 2 | 0 | 20 | 16 | 40 | 32 | 12 | 59 | 38 | 1 | 8 | 1 | 1 | 2 | 3 |
|  |  | T3 | 128.294 | 34.8162 | -22.0 | 6 | 12 | 0 | 0 | 74 | 89 | 70 | 70 | 14 | 119 | 38 | 0 | 6 | 1 | 1 | 1 | 6 |
|  |  | T4 | 128.303 | 34.5857 | -22.0 | 2 | 2 | 1 | 1 | 16 | 33 | 20 | 22 | 7 | 43 | 16 | 1 | 1 | 1 | 1 | 1 | 2 |
|  |  | T5 | 128.133 | 34.8052 | -21.5 | 5 | 9 | 2 | 0 | 29 | 68 | 33 | 40 | 1 | 61 | 1 | 1 | 1 | 1 | 1 | 1 | 9 |
|  |  | T6 | 128.133 | 34.6872 | -19.7 | 3 | 0 | 1 | 0 | 21 | 36 | 28 | 31 | 13 | 74 | 23 | 1 | 1 | 1 | 1 | 1 | 5 |
|  |  | T7 | 128.137 | 34.5765 | -20.9 | - | - | - | - | - | - | - | - | - | - | - | - | - | - | - | - | - |
|  |  | T8 | 128.017 | 34.6324 | -22.2 | 4 | 11 | 5 | 0 | 72 | 56 | 65 | 52 | 32 | 113 | 83 | 1 | 30 | 57 | 24 | 7 | 9 |
|  |  | T9 | 128.023 | 34.5500 | -19.1 | 2 | 3 | 1 | 1 | 23 | 37 | 37 | 20 | 12 | 129 | 19 | 1 | 2 | 1 | 1 | 1 | 2 |
|  |  | T10 | 127.833 | 34.7119 | -21.5 | 0 | 1 | 2 | 1 | 21 | 1 | 62 | 21 | 1 | 54 | 1 | 1 | 2 | 0 | 1 | 0 | 3 |
|  |  | T11 | 127.833 | 34.4595 | -21.4 | 1 | 1 | 2 | 1 | 26 | 40 | 36 | 29 | 16 | 41 | 28 | 1 | 2 | 1 | 1 | 1 | 2 |
|  |  | T12 | 127.842 | 34.5775 | -20.1 | 3 | 1 | 1 | 1 | 24 | 29 | 33 | 42 | 6 | 42 | 12 | 1 | 1 | 1 | 1 | 1 | 4 |
| Aug.23-27, 2019 | Non-patch area | T1 | 128.418 | 34.7010 | -20.7 | 11 | 1 | 3 | 1 | 65 | 50 | 70 | 51 | 27 | 125 | 36 | 4 | 18 | 25 | 4 | 3 | 5 |
|  |  | T2 | 128.418 | 34.5939 | -21.8 | 10 | 5 | 5 | 1 | 71 | 31 | 82 | 69 | 39 | 71 | 87 | 1 | 42 | 10 | 14 | 4 | 5 |
|  |  | T3 | 128.294 | 34.8162 | -20.0 | 5 | 0 | 3 | 1 | 75 | 77 | 49 | 36 | 44 | 119 | 71 | 3 | 7 | 8 | 20 | 4 | 3 |
|  |  | T4 | 128.303 | 34.5857 | -19.3 | 10 | 13 | 4 | 0 | 58 | 65 | 54 | 44 | 24 | 69 | 43 | 2 | 16 | 1 | 6 | 3 | 10 |
|  |  | T5 | 128.133 | 34.8052 | -18.9 | 11 | 2 | 7 | 1 | 92 | 67 | 92 | 66 | 40 | 74 | 75 | 2 | 19 | 9 | 13 | 4 | 5 |
|  |  | T6 | 128.133 | 34.6872 | -20.7 | 6 | 4 | 1 | 1 | 60 | 50 | 59 | 44 | 28 | 55 | 29 | 2 | 18 | 2 | 9 | 5 | 5 |
|  |  | T7 | 128.137 | 34.5765 | -21.9 | 4 | 2 | 2 | 6 | 49 | 57 | 49 | 62 | 35 | 118 | 23 | 4 | 4 | 15 | 15 | 8 | 3 |
|  |  | T8 | 128.017 | 34.6324 | -19.3 | 14 | 3 | 4 | 6 | 65 | 69 | 62 | 81 | 25 | 118 | 35 | 2 | 10 | 4 | 7 | 2 | 6 |
|  |  | T9 | 128.023 | 34.5500 | -21.0 | 6 | 2 | 1 | 1 | 61 | 79 | 55 | 30 | 16 | 87 | 47 | 0 | 19 | 3 | 2 | 9 | 6 |
|  |  | T10 | 127.833 | 34.7119 | -20.6 | 7 | 5 | 11 | 4 | 81 | 42 | 86 | 46 | 42 | 52 | 91 | 0 | 53 | 1 | 18 | 1 | 9 |
|  |  | T11 | 127.833 | 34.4595 | -19.9 | 11 | 14 | 5 | 1 | 73 | 86 | 67 | 39 | 26 | 87 | 79 | 2 | 21 | 10 | 41 | 2 | 16 |
|  | Patch area | T12 | 127.842 | 34.5775 | -18.2 | 5 | 3 | 4 | 1 | 87 | 97 | 198 | 113 | 78 | 283 | 65 | 5 | 27 | 11 | 45 | 7 | 14 |
| Sep.19, 2019 | Non-patch area | S1 | 128.343 | 34.6535 | -22.1 | 8 | 1 | 2 | 1 | 35 | 36 | 23 | 19 | 12 | 42 | 7 | 0 | 3 | 1 | 17 | 3 | 2 |
|  |  | S2 | 128.34 | 34.6709 | -21.1 | 3 | 2 | 1 | 2 | 14 | 27 | 15 | 26 | 9 | 48 | 6 | 0 | 14 | 12 | 1 | 4 | 3 |
|  |  | S3 | 128.341 | 34.7067 | -21.0 | 4 | 3 | 2 | 3 | 18 | 36 | 16 | 25 | 12 | 52 | 5 | 0 | 22 | 10 | 3 | 5 | 5 |
|  |  | S4 | 128.352 | 34.7350 | -21.9 | 4 | 2 | 2 | 1 | 18 | 32 | 18 | 25 | 11 | 46 | 6 | 1 | 23 | 10 | 2 | 5 | 4 |
|  |  | S5 | 128.341 | 34.7663 | -21.5 | 4 | 3 | 2 | 1 | 32 | 47 | 38 | 37 | 16 | 63 | 14 | 2 | 6 | 1 | 5 | 5 | 5 |
|  |  | S6 | 128.392 | 34.7753 | -21.0 | 4 | 4 | 2 | 1 | 26 | 40 | 26 | 31 | 14 | 49 | 11 | 0 | 4 | 0 | 2 | 5 | 7 |
|  | Patch area | S7 | 128.375 | 34.7663 | -21.5 | 4 | 3 | 2 | 2 | 24 | 38 | 26 | 29 | 13 | 80 | 13 | 0 | 3 | 1 | 5 | 6 | 5 |
|  |  | S8 | 128.362 | 34.7890 | -22.0 | 4 | 2 | 2 | 1 | 24 | 32 | 26 | 29 | 13 | 123 | 12 | 2 | 3 | 7 | 2 | 4 | 3 |
|  |  | S9 | 128.385 | 34.7539 | -21.2 | 3 | 1 | 1 | 2 | 22 | 39 | 26 | 33 | 7 | 73 | 6 | 0 | 3 | 0 | 7 | 2 | 3 |
|  |  | S10 | 128.361 | 34.8070 | -21.7 | 3 | 2 | 2 | 2 | 22 | 25 | 27 | 29 | 15 | 92 | 8 | 2 | 5 | 1 | 19 | 4 | 3 |
|  |  | S11 | 128.38 | 34.7853 | -20.4 | 7 | 2 | 2 | 2 | 38 | 61 | 47 | 38 | 24 | 94 | 0 | 0 | 20 | 1 | 13 | 8 | 5 |

**Table S3.** The concentrations of chlorophyll *a*, peridinin, and fucoxanthin in seawaters off Tongyeong, Korea　in September 19, 2019.

| Station | | | | | Pigments (μg L^-1^) | | |
| --- | --- | --- | --- | --- | --- | --- | --- |
|  | Type | St. | Longitude | Latitude | Chlorophyll *a* | Peridinin | Fucoxanthin |
| Sep.19, 2019 | Non-patch area | S1 | 128.343 | 34.6535 | 0.3 | 0.2 | 0.4 |
|  |  | S2 | 128.34 | 34.6709 | 0.1 | 0 | 0.1 |
|  |  | S3 | 128.341 | 34.7067 | 0.6 | 0.6 | 1.5 |
|  |  | S4 | 128.352 | 34.7350 | 0.2 | 0.1 | 0.2 |
|  |  | S5 | 128.341 | 34.7663 | 0.4 | 0.5 | 0.4 |
|  |  | S6 | 128.392 | 34.7753 | 1.4 | 1.2 | 0.1 |
|  | Patch area | S7 | 128.375 | 34.7663 | 0.9 | 1.2 | 0.4 |
|  |  | S8 | 128.362 | 34.7890 | 1.9 | 1.3 | 0.5 |
|  |  | S9 | 128.385 | 34.7539 | 2.3 | 3.3 | 0.2 |
|  |  | S10 | 128.361 | 34.8070 | 1.8 | 2.7 | 0.3 |
|  |  | S11 | 128.38 | 34.7853 | 1.1 | 2.1 | 0.1 |
